# Supplementary material for: Human spinal cord in vitro differentiation pace is initially maintained in heterologous embryonic environments
Source: eLife. 2022 Feb 21;11:e67283. doi: 10.7554/eLife.67283 (PMC8929931; doi:10.7554/eLife.67283)
Supplement: Transparent reporting form [file elife-67283-transrepform1.docx]

***eLife’s* transparent reporting form**

We encourage authors to provide detailed information *within their submission* to facilitate the interpretation and replication of experiments. Authors can upload supporting documentation to indicate the use of appropriate reporting guidelines for health-related research (see [EQUATOR Network](http://www.equator-network.org/%20)), life science research (see the [BioSharing Information Resource](https://biosharing.org/)), or the [ARRIVE guidelines](http://www.plosbiology.org/article/info:doi/10.1371/journal.pbio.1000412) for reporting work involving animal research. Where applicable, authors should refer to any relevant reporting standards documents in this form.

If you have any questions, please consult our Journal Policies and/or contact us: [editorial@elifesciences.org](mailto:editorial@elifesciences.org).

**Sample-size estimation**

- You should state whether an appropriate sample size was computed when the study was being designed
- You should state the statistical method of sample size computation and any required assumptions
- If no explicit power analysis was used, you should describe how you decided what sample (replicate) size (number) to use

Please outline where this information can be found within the submission (e.g., sections or figure legends), or explain why this information doesn’t apply to your submission:

To ensure statistical analysis could be carried out a minimum of 3 independent (biological) replicates were generated for all human pluripotent stem cell differentiations. This is accepted practice in the field. For rosette grafting experiments rosettes from 3 independent experiments were grafted on 3 different days. Samples of human embryonic spinal cord are rare samples provided by the UK Human Developmental Biology Resource. We were provided with 3 tissue samples at different stages CS12, CS13 and CS18 and sections of brachial spinal cord from one CS13 and one CS16 embryo. Sample size information can be found in the Materials and Methods and relevant figure legends.

**Replicates**

- You should report how often each experiment was performed
- You should include a definition of biological versus technical replication
- The data obtained should be provided and sufficient information should be provided to indicate the number of independent biological and/or technical replicates
- If you encountered any outliers, you should describe how these were handled
- Criteria for exclusion/inclusion of data should be clearly stated
- High-throughput sequence data should be uploaded before submission, with a private link for reviewers provided (these are available from both GEO and ArrayExpress)

Please outline where this information can be found within the submission (e.g., sections or figure legends), or explain why this information doesn’t apply to your submission:

All cell counts are an average percentage of cells positive for each protein of interest compared to total (DAPI+ or GFP+ve) cells counted within a field of neural progenitors (D4) or a rosette and contacting cells at its periphery. Grafted chicken embryos: n= at least 3 grafted chicken embryos (biological replicates) n= 3 transverse sections (technical replicates) were used to determine the proportion of cells expressing a protein of interest. Transplanted human cells were only counted when cells were positive for GFP, immunoreactivity for protein of interest and DAPI. To avoid counting the same cells in consecutive sections, alternate sections were used for each immunolabelling experiment. This information is provided in the Materials and Methods and relevant figure legends.

**Statistical reporting**

- Statistical analysis methods should be described and justified
- Raw data should be presented in figures whenever informative to do so (typically when N per group is less than 10)
- For each experiment, you should identify the statistical tests used, exact values of N, definitions of center, methods of multiple test correction, and dispersion and precision measures (e.g., mean, median, SD, SEM, confidence intervals; and, for the major substantive results, a measure of effect size (e.g., Pearson's r, Cohen's d)
- Report exact p-values wherever possible alongside the summary statistics and 95% confidence intervals. These should be reported for all key questions and not only when the p-value is less than 0.05.

Statistical analysis was performed using non-parametric Mann-Whitney U test for none normally distributed data with PRISM V8. All cell counts are an average percentage of cells positive for each protein of interest compared to total (DAPI+ or GFP+) cells counted. Results are represented as means +/- SEM or SD and p-values are indicated in figures. This information is provided in the Materials and Methods and relevant figure legends.

Please outline where this information can be found within the submission (e.g., sections or figure legends), or explain why this information doesn’t apply to your submission:

(For large datasets, or papers with a very large number of statistical tests, you may upload a single table file with tests, Ns, etc., with reference to sections in the manuscript.)

**Group allocation**

- Indicate how samples were allocated into experimental groups (in the case of clinical studies, please specify allocation to treatment method); if randomization was used, please also state if restricted randomization was applied
- Indicate if masking was used during group allocation, data collection and/or data analysis

Please outline where this information can be found within the submission (e.g., sections or figure legends), or explain why this information doesn’t apply to your submission:

In every graph presented, each dot represents data from a single defined field of cells at D4 or a single rosette at D6 and later for in vitro analyses.
For grafted chicken embryos, each dot represents data from one histological transverse section through a grafted rosette. These data can be found in the Materials and Methods and relevant figure legends.

**Additional data files (“source data”)**

- We encourage you to upload relevant additional data files, such as numerical data that are represented as a graph in a figure, or as a summary table
- Where provided, these should be in the most useful format, and they can be uploaded as “Source data” files linked to a main figure or table
- Include model definition files including the full list of parameters used
- Include code used for data analysis (e.g., R, MatLab)
- Avoid stating that data files are “available upon request”

Please indicate the figures or tables for which source data files have been provided:

Figures 1,2,4, 4S1, 4S4, 5,6,7,8,9,
